# Association of quarterly prevalence of e‐cigarette use with ever regular smoking among young adults in England: a time–series analysis between 2007 and 2018

**DOI:** 10.1111/add.15838

**Published:** 2022-03-09

**Authors:** Emma Beard, Jamie Brown, Lion Shahab

**Affiliations:** ^1^ Department of Behavioural Science and Health University College London London UK; ^2^ SPECTRUM Consortium, Department of Behavioural Science and Health University College London London UK

**Keywords:** ARIMAX, e‐cigarettes, England, smoking, Smoking Toolkit Study, time–series

## Abstract

**Aims:**

To assess how changes in the prevalence of e‐cigarette use among young adults have been associated with changes in the uptake of smoking in England between 2007 and 2018.

**Design:**

Time–series analysis of population trends with autoregressive integrated moving average with exogeneous input (ARIMAX models).

**Setting:**

England.

**Participants:**

Data were aggregated quarterly on young adults aged 16–24 years (*n* = 37 105) taking part in the Smoking Toolkit Study.

**Measures:**

In the primary analysis, prevalence of e‐cigarette use was used to predict prevalence of ever regular smoking among those aged 16–24. Sensitivity analyses stratified the sample into those aged 16–17 and 18–24. Bayes’ factors and robustness regions were calculated for non‐significant findings [effect size beta coefficient (B) = 3.1].

**Findings:**

There was evidence for no association between the prevalence of e‐cigarette use and ever regular smoking among those aged 16–24 [B = –0.015, 95% confidence interval (CI) = –0.046 to 0.016; *P* = 0.341; Bayes factor (BF) = 0.002]. Evidence for no association was also found in the stratified analysis among those aged 16–17 (B = 0.070, 95% CI –0.014 to 0.155, *P* = 0.102; BF = 0.015) and 18–24 (B = –0.021, 95% CI –0.053 to 0.011; *P* = 0.205; BF = 0.003). These findings were able to rule out percentage point increases or decreases in ever regular smoking prevalence greater than 0.31% or less than −0.03% for 16–17‐year‐olds and 0.01 or −0.08% for 18–24‐year‐olds for every 1%‐point increase in e‐cigarette prevalence.

**Conclusion:**

Prevalence of e‐cigarette use among the youth population in England does not appear to be associated with substantial increases or decreases in the prevalence of smoking uptake. Small associations cannot be ruled out.

## INTRODUCTION

Policymakers have cited a gateway from e‐cigarette use to cigarette smoking as a key motivation for various regulations to heavily restrict e‐cigarettes. For example, India announced an executive order in 2019 to ban the production, import and sale of electronic cigarettes, in part due to the argument that ‘when used by never smokers, these devices can be a gateway for cigarette smoking and other drug use’ [1]. The US Food and Drug Administration (FDA) announced a ban on non‐tobacco‐flavoured products saying that ‘we will not stand idly by as [e‐cigarettes] become an on‐ramp to combustible cigarettes’ [2]. However, there remains academic debate on whether there is good evidence that e‐cigarettes act as a gateway into smoking [3, 4, 5].

Longitudinal observational studies show that there is a strong association between initiation of e‐cigarette use and later use of cigarettes, including among people who have never smoked [6, 7, 8]. However, these findings may reflect a common liability whereby people with characteristics or a social environment that make them susceptible to use e‐cigarettes are also more likely to smoke cigarettes later [9].

One way to rule out this self‐selection bias is with a randomized controlled trial. However, it is unethical and impracticable to allocate never smokers to e‐cigarette or no e‐cigarette use. Population time–series data offer an alternative option. These can rule out such selection bias, as analyses are at the population rather than individual level [10, 11, 12]. Time–series analysis can assess the gateway effect by looking at the association between the prevalence of e‐cigarette use among young adults with uptake of smoking generally and among never smokers in particular. If gateway effects existed, we would expect that an increase in e‐cigarette prevalence would be associated with an increase in the prevalence of smoking uptake. Time–series models are particularly suited to address this question. First, time–series models go beyond simple correlation and assess the extent to which the past prevalence of a series (e.g. e‐cigarette use) can forecast the future prevalence of another series (e.g. smoking uptake) [13, 14]. Secondly, population‐level time–series analysis has the advantage of estimating directly the net population‐level effect of e‐cigarettes. For example, a gateway could act through e‐cigarette use by an individual leading them to start smoking, but could also occur if the promotion and presence of e‐cigarettes on the UK market re‐normalizes smoking. This effect could only be detected at a population level. The recent increases in the use of e‐cigarettes by young people provides a useful natural experiment [15].

This study used time–series analysis to assess if a gateway effect exists at a population level by determining if there is any association between the prevalence of current use of e‐cigarettes with prevalence of ever regular smoking (as a measure of uptake) among those aged 16–24 years in England. To our knowledge, this is the first study to use a time–series methodology to address this question and follows a call for methodological triangulation to draw causal inference regarding gateway effects [16].

## METHODS

### Sample size

The sample size of this study meets previous requirements of at least 50 time–points and at least 2 years of data collection to account for seasonality [17]. We have shown previously, using power simulations, that the Smoking Toolkit Study (STS) data set in England has 80% power to detect a 0.1% in smoking behaviour as a consequence of a 1% change in e‐cigarette prevalence [18].

### Design

Data on the explanatory and outcome variables come from the STS. The STS is a monthly survey of a representative sample of the population in England aged 16+ [19]. The STS was established in 2006 and involves monthly household surveys using a form of random location sampling, with initial random selection of grouped output areas (containing ~ 300 households), stratified by socio‐demographic characteristic and region. Interviewers then select houses within these areas that are most likely to fulfil quotas and conduct face‐to‐face computer‐assisted interviews with one member per household. The quotas are tailored to the output area and the probability of certain groups being at home. Participants from the STS appear to be representative of the population in England, having similar socio‐demographic composition as other large national surveys, such as the Health Survey for England, and comparisons with sales data suggest representative estimates of cigarette consumption [20]. Data on the covariate mass media expenditure were obtained from Public Health England and the affordability index calculated using data from the Office for National Statistics and the STS [21].

### Measures

Output variable: prevalence of ever regular smoking among 16–24‐year‐olds

Ever regular smoking among 16–24‐year‐olds is a widely used indicator of uptake [22, 23, 24, 25]. While the ever‐smoking rate in young adults in a given quarter does not provide a direct and absolute measure of uptake in that quarter, changes in this figure quarter on quarter provide a population‐level indication of changes in uptake. Uptake after the age of 24 is rare, so ever regular smoking up to this age should capture almost all uptake [22, 26]. The prevalence of ever regular smoking was calculated as the proportion of all participants aged 16–24 who reported that they smoked cigarettes (including hand‐rolled) every day; that they smoked cigarettes (including hand‐rolled), but not every day; that they did not smoke cigarettes at all, but smoked tobacco of some kind (e.g. cigar); that they have stopped smoking completely in the last year; or that they had stopped smoking completely more than a year ago. Those counted as never smokers responded: ‘I have never been a smoker (i.e. smoked for a year or more)’.

### Input variable: prevalence of e‐cigarette use among 16–24‐year‐olds

Participants who reported that they smoked cigarettes were then asked the following questions, with one of the response options being e‐cigarettes:

1. ‘Which, if any, of the following are you currently using to help you cut down the amount you smoke?’
2. ‘Do you regularly use any of the following in situations when you are not allowed to smoke?’

Current smokers and those who had stopped smoking in the last year (past‐year smokers) were also asked:

3. ‘Can I check, are you using any of the following either to help you stop smoking, to help you cut down or for any other reason at all?’

All other participants (non‐smokers) were asked:

4. ‘Can I check, are you using any of the following?’

Prevalence of use of e‐cigarettes was obtained for each quarter by counting the number of respondents who answered ‘electronic cigarette’ in response to any of the four questions above, divided by the total sample size in that quarter. As a sensitivity analysis, we also include the prevalence of e‐cigarette use among never smokers as an input variable. This was obtained for each quarter by counting the number never smokers who answered ‘electronic cigarettes’, divided by the total sample size in that quarter of never smokers.

### Data on covariates

A number of tobacco control policies were adjusted for in the analyses using a composite score, coded as 0 when no policies were present and 1 for the quarter when policies were present (reflecting a pulse effect). These include the move in commissioning of stop smoking services to local authorities in April 2013 [27], introduction of a smoking ban in July 2007 [28], licensing of nicotine replacement therapy (NRT) for harm reduction in December 2009 [29], change in the minimum age of sale of cigarettes October 2007 [30] and the tobacco products directive/plain packaging in May 2016 [31]. An affordability index was derived using a modified formula published by the Office for National Statistics [21, 32]: $\frac{\text{adjusted real household}s^{\prime }\text{disposable income index}}{\text{weekly tobacco expenditure index}}\;x\;100$.

An affordability index greater than 100 in a given quarter signifies that cigarettes were more affordable than in the first quarter of 2007 [33].

Monthly tobacco mass media expenditure (in £million) was also adjusted for and was obtained from Public Health England. Total spending on campaigns was calculated for each quarter and included spending on ‘Smokefree’ campaigns, Stoptober campaigns and Health Harms campaigns. Spend included TV, radio, print, cinema and on‐line advertisements.

### Missing data

Prevalence of e‐cigarette use among never and long‐term ex‐smokers was only available from October 2013. As use in these groups was extremely rare immediately after October 2013, it was assumed that use before October 2013 was zero.

Data were also only available on the prevalence of use of e‐cigarettes among smokers from April 2011. Prevalence of e‐cigarette use between January 2007 and April 2011 was assumed to be 0.1% of smokers based on other surveys [34]. Two waves of data were collected in March 2007; these were combined. No data were collected in December 2008. Variables during this period were calculated as an average of the quarter before and the quarter after. Data on e‐cigarette use among past‐year smokers were not recorded for 6 months (May 2012, July 2012, September 2012, November 2012, January 2013, March 2013). Quarters covering these months were estimated instead based on available months.

### Analysis

The analysis plan (see on‐line Supporting information) and data set were pre‐registered on the Open Science Framework (https://osf.io/8b7pr). One major amendment was the exclusion of affordability as a covariate due to evidence of high multi‐collinearity and model instability when it was included in the models. Results including affordability can be found in the Supporting information for affordability. In Supporting information, Table S1a a lag of one is used for mass media, and in Supporting information, Table S1b no lag is used. Comparison of transfer functions using the Aikaike information criterion (AIC) showed that a lag of one provided the best model fit. Supporting information, Table S2 shows that including affordability did not increase model fit substantially. The analysis plan also stated that we would use data from November 2006 when the STS was established, but this was changed to January 2007 when data on mass media spend first became available. All data were analysed in R and aggregated quarterly. Strengthening the reporting of observational studies in epidemiology (STROBE) guidelines were followed [35]. All analyses were two‐tailed, reflecting the null hypothesis that the association between e‐cigarette use and smoking behaviour could be positive or negative.

### Primary analysis

The primary analysis focused upon the association between e‐cigarette prevalence and ever regular smoking prevalence among those aged 16–24 years, with an additional stratified analysis with those aged 16–17 and 18–24. Autoregressive integrated moving average with exogeneous input (ARIMAX) analysis was used to assess the association between changes in the prevalence of e‐cigarette use and prevalence of ever regular smoking as a measure of uptake. Standard recommended procedures were followed. This included assessing the series for outlying values and the presence of exogeneity using the Granger causality test. Unit root tests, the cross‐correlation function and the autocorrelation function were used to identify the level of differencing, specification of the transfer function (lags between the series) and the presence of autoregressive and moving average autocorrelation.

Bayes factors were calculated for non‐significant findings. Bayes factors help to determine if there is evidence for the null hypothesis of no difference or if the data are insensitive to detect an effect. This approach requires the specification of an expected effect size (i.e. a plausible range of predicted values based on previous studies, judgement or clinical significance), the published effect size (e.g. risk difference) and standard error of this parameter. It assumes that the sampling distribution of the parameter estimate is Gaussian. The expected effect size used in the Bayes factor calculation was derived from a recent meta‐analysis of seven cohort studies in youths (B = 3.1) [8]. A Gaussian distribution was specified where the population parameter values close to the mean are assumed to be more plausible than others. A default standard deviation of mean/2 is often used. Bayes factors are interpreted based on Jeffreys’ cut‐offs, which indicate the strength of evidence for or against the null hypothesis [36]. We also calculated a robustness region for each Bayes factor.

### Sensitivity analysis: robustness of data assumptions

We conducted several sensitivity analyses to test the robustness of two different data assumptions. In the primary analysis, we assumed that e‐cigarette use among respondents who were never smokers and long‐term ex‐smokers was zero before October 2013. In the first sensitivity analysis, we adjusted the ARIMAX model for a step level change in October 2013. Next, we used a ratio of use among long‐term ex‐smokers and never smokers versus past‐year smokers for the first year of assessment to adjust the prevalence prior to October 2013. Another assumption for the primary analysis was that prevalence of e‐cigarette use between January 2007 and April 2011 was 0.1%, when questions on e‐cigarettes were not asked of anyone. In sensitivity analyses we instead (a) assumed a linear function starting at close to zero from 2007 and (b) used Kalman smoothing for univariate time–series to impute the values.

### Sensitivity analysis: prevalence among never smokers and ever regular smoking

The next sensitivity analysis involved restricting the input variable to e‐cigarette prevalence among never smokers to provide a more stringent test of the on‐ramp gateway hypothesis.

### Sensitivity analysis: feedback from prevalence of ever regular smoking

Due to evidence of violation of the assumption of weak exogeneity for affordability, and it being theoretically plausible that ever regular smoking may affect prevalence of e‐cigarette use, additional structural time–series models known as structural vector autoregression (SVAR) were run.

### Patient involvement

No patients were involved in setting the research question or the outcome measures, nor were they involved in developing plans for recruitment, design, or implementation of the study. No patients were asked to advise on interpretation or writing up of results. There are no plans to disseminate the results of the research directly to study participants or any specific patient community.

### Ethical approval

Ethical approval for the STS was granted by the UCL ethics committee (ID 0498/001). Ethical approval was not required for use of data from stop smoking services, as the data are publicly available. Data sets are available on the Open Science Framework (https://osf.io/8b7pr).

## RESULTS

**FIGURE 1:**
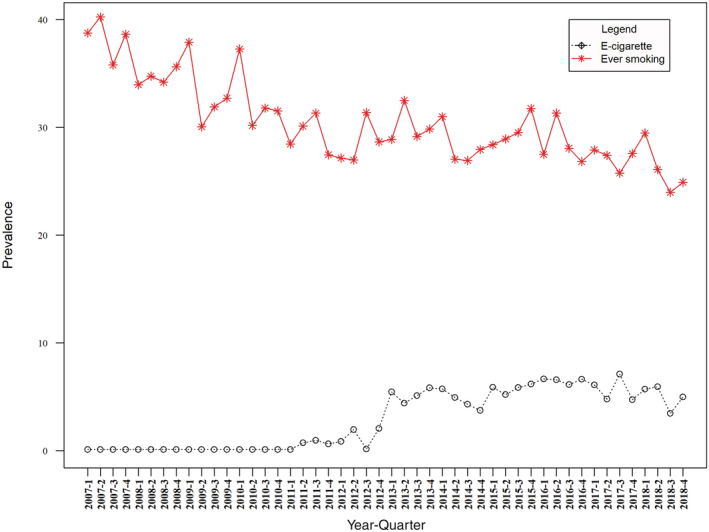
Prevalence of ever regular smoking and e‐cigarette use over the study period among those aged 16–24 years

Data were collected on 37 105 participants aged 16–24 between January 2007 and December 2018. Of these, 30.5% [95% confidence interval (CI) = 30.0–31.0] were ever regular smokers and 27.4% (95% CI = 27.0–27.9) were past‐year smokers. Table 1 and Fig. 1 show the descriptive statistics for the time–series.

**TABLE 1:** Mean, standard deviation (SD), 95% confidence interval (CI) start and end values of the time–series (per quarter) for prevalence of e‐cigarette use and ever regular smoking prevalence

			95% CI			
Time–series	Mean	SD	Lower	Upper	Start	End
Primary analysis						
E‐cigarette prevalence aged 16–24	2.9	2.7	2.1	3.7	0.1	5.0
Ever regular smoking prevalence aged 16–24	30.5	3.9	29.4	31.6	38.8	24.9
Sensitivity analysis						
E‐cigarette prevalence aged 16–17	1.8	2.2	1.2	2.4	0.1	3.6
Ever regular smoking prevalence aged 16–17	16.9	5.6	15.3	18.5	29.3	15.4
E‐cigarette prevalence aged 18–24	3.1	2.8	2.3	3.9	0.1	5.1
Ever regular smoking prevalence aged 18–24	32.9	4.3	31.7	34.1	40.9	25.8
E‐cigarette prevalence aged 16–24 with ratio correction for missing data	3.0	2.7	2.2	3.8	0.1	5.0
E‐cigarette prevalence aged 16–24 with linear function for missing data	3.0	2.6	2.3	3.7	0.0	5.0
E‐cigarette prevalence aged 16–24 with Kalman smoothing for missing data	3.2	2.4	2.5	3.9	0.8	5.0
E‐cigarette prevalence aged 16–24 among never smokers	0.2	0.2	0.1	0.3	0.1	1.0
E‐cigarette prevalence aged 16–17 among never smokers	0.3	0.7	0.1	0.5	0.1	3.4
E‐cigarette prevalence 18–24 among never smokers	0.2	0.2	0.1	0.3	0.1	0.7

### Primary analysis

There was evidence of no association between the prevalence of e‐cigarette use and ever regular smoking among those aged 16–24 in both the unadjusted (B = –0.013, 95% CI = –0.046 to 0.021, *P* = 0.461; BF = 0.022) and adjusted models B = –0.015, 95% CI = –0.046 to 0.016, *P* = 0.341; BF 0.002) (see Tables 2 and 3). Bayes factor robustness regions suggest that the null hypothesis was more likely than, on average, a 1% percentage point increase in the prevalence of e‐cigarette use being associated with more than a 0.014 percentage point increase in ever regular smoking among 16–24‐year‐olds. Conversely, negative associations less than −0.058 could be ruled out.

**TABLE 2:** Primary analysis—unadjusted estimated percentage point changes in ever regular smoking prevalence as a function of e‐cigarette prevalence, based on autoregressive integrated moving average with exogeneous input (ARIMAX)

	Unadjusted								
	Prevalence of ever regular smoking, aged 16–24			Prevalence of ever regular smoking, aged 16–17			Prevalence of ever regular smoking, aged 18–24		
	Percentage change per 1% change in the exposure	95% CI	*P*‐value	Percentage change per 1% change in the exposure	95% CI	*P*‐ value	Percentage change per 1% change in the exposure	95% CI	*P*‐value
Prevalence of current e‐cigarette use aged 16–24 Prevalence of current e‐cigarette use aged 16–17 Prevalence of current e‐cigarette use aged 18–24	–0.013	−0.046 to 0.021	0.461	0.077	−0.006 to 0.159	0.068	−0.018	−0.052 to 0.016	0.301
Model Adjusted R‐squared	ARIMA (0, 1, 1) (0, 0, 0)_4_ 0.66			ARIMA (0, 1, 1) (0, 0, 0)_4_ 0.37			ARIMA (0, 1, 1) (0, 0, 0)_4_ 0.62		
Bayes factor (robustness region)	0.002 (−∞ to −0.052 ∪ 0.018 to ∞)			0.022 (−∞ to −0.025 ∪ 0.400 to ∞)			0.003 (−∞ to −0.065 ∪ 0.015 to ∞)		

CI = confidence interval.

**TABLE 3:** Primary analysis—adjusted estimated percentage point changes in ever regular smoking prevalence as a function of e‐cigarette prevalence, based on autoregressive integrated moving average with exogeneous input (ARIMAX)

	Adjusted								
	Prevalence of ever regular smoking aged 16–24			Prevalence of ever regular smoking aged 16–17			Prevalence of ever regular smoking aged 18–24		
	Percentage change per 1% change in the exposure	95% CI	*P*‐value	Percentage change per 1% change in the exposure	95% CI	*P*‐value	Percentage change per 1% change in the exposure	95% CI	*P*‐value
Prevalence of current e‐cigarette use aged 16–24 Prevalence of current e‐cigarette use aged 16–17 Prevalence of current e‐cigarette use aged 18–24	−0.015	−0.046 to 0.016	0.341	0.070	−0.014 to 0.155	0.102	−0.021	−0.053 to 0.011	0.205
Tobacco control mass media spend	0.018	−0.001 to 0.038	0.069	−0.004	−0.084 to 0.077	0.930	0.020	−0.002 to 0.042	0.080
	Total change due to the exposure	95% CI	*P*‐value	Total change due to the exposure	95% CI	*P*‐value	Total change due to the exposure	95% CI	*P*‐value
Tobacco control policies	0.061	0.001 to 0.121	0.047	0.086	−0.171 to 0.343	0.512	0.054	−0.015 to 0.123	0.122
Model Adjusted R‐squared	ARIMA (0, 1, 1) (0, 0, 0)_4_ 0.70			ARIMA (0, 1, 1) (0, 0, 0)_4_ 0.38			ARIMA (0, 1, 1) (0, 0, 0)_4_ 0.65		
Bayes factor (robustness region)	0.022 (−∞ to −0.058 ∪ 0.014 to ∞)			0.015 (−∞ to −0.028 ∪ 0.310 to ∞)			0.003 (−∞ to −0.080 ∪ 0.013 to ∞)		

CI = confidence interval.

After stratifying the results for those aged 16–17 and 18–24, there was no association in the unadjusted and adjusted sensitivity analysis between prevalence of e‐cigarette use and ever regular smoking among these (see Tables 2 and 3). Bayes factor robustness regions suggest that the null hypothesis was more likely than, on average, a 1% increase in the prevalence of e‐cigarette use being associated with more than a 0.310 percentage point increase in ever regular smoking among 16–17‐year‐olds and a 0.013 percentage point increase in ever regular smoking among 18–24‐year‐olds. Conversely, respective negative associations less than −0.028 and −0.080 percentage points could also be ruled out.

### Sensitivity analysis: testing the robustness of data assumptions

Similar findings were found for the association between e‐cigarette prevalence among 16–24‐year‐olds and ever regular smoking prevalence when additionally adjusting for a step level change in October 2013 (B_adjusted_ = −0.010, 95% CI= –0.035 to 0.014; *P* = 0.411). Using a ratio of use among long‐term ex‐smokers and never smokers versus past‐year smokers for the first year of assessment to adjust the prevalence prior to October 2013 also did not meaningfully change the findings (B_unadjusted_ = −0.010, 95% CI = –0.048 to 0.027; *P* = 0.579; B_adjusted_ = −0.014, 95% CI = –0.049 to 0.020; *P* = 0.415).

Similar estimates were found using different assumptions for missing data between January 2009 and April 2011. When assuming a linear function, there was no significant association between prevalence of e‐cigarettes and prevalence of ever regular smoking among those aged 16–24 (B_unadjusted_ = −0.016, 95% CI = –0.041 to 0.009; *P* = 0.203; B_adjusted_ = −0.017, 95% CI = –0.041 to 0.006; *P* = 0.143). The same was true when Kalman smoothing was used to impute values (B_unadjusted_ = −0.012, 95% CI = –0.055 to 0.032; *P* = 0.597; B_adjusted_ = −0.013, 95% CI = –0.054 to 0.027; *P* = 0.515).

### Sensitivity analysis: e‐cigarette prevalence among never smokers and ever regular smoking

There was no significant association between prevalence of e‐cigarette use and ever regular smoking among those aged 16–24 when e‐cigarette use was restricted to never smokers (B_unadjusted_ = −0.017, 95% CI = –0.057 to 0.0.023; *P* = 0.394; B_adjusted_ = −0.019, 95% CI = –0.057 to 0.019; *P* = 0.321). Prevalence of e‐cigarette use among never smokers was also not associated with prevalence of ever regular smoking either among those aged 16–17 (B_unadjusted_ = 0.052, 95% CI = –0.044 to 0.147; *P* = 0.288; B_adjusted_ = 0.055, 95% CI = –0.041 to 0.151; *P* = 0.262) or those aged 18–24 (B_unadjusted_ = −0.034, 95% CI = –0.081 to 0.013; *P* = 0.155; B_adjusted_ = −0.036, 95% CI = –0.081 to 0.009; *P* = 0.116).

### Sensitivity analysis: accounting for feedback from prevalence of ever regular smokin*g*

In the unadjusted and adjusted SVAR models (see Table 4 and Supporting information, Table S3), there was an immediate effect (short‐term impact) of the prevalence of e‐cigarette use among those aged 16–17 on ever regular smoking prevalence in the same age group. The impulse response function (IRF) can be interpreted as a 1% increase in the prevalence of e‐cigarette use being associated with an immediate 0.097 percentage point increase in the prevalence of ever regular smoking. There were no significant associations between prevalence of e‐cigarettes and ever regular smoking for those aged 16–24 and 18–24.

**TABLE 4:** Unadjusted estimated percentage point changes in ever regular smoking prevalence as a function of e‐cigarette prevalence, based on structural vector autoregression (SVAR)

	Prevalence of ever regular smoking aged 16–24			Prevalence of ever regular smoking aged 16–17			Prevalence of ever regular smoking, aged 18–24		
	IRF Percentage change per 1% change in the exposure	95% CI	*P*‐value	IRF Percentage change per 1% change in the exposure	95% CI	*P*‐value	IRF Percentage change per 1% change in the exposure	95% CI	*P*‐value
Unadjusted									
Prevalence of current e‐cigarette use aged 16–24 Prevalence of current e‐cigarette use aged 16–17 Prevalence of current e‐cigarette use aged 18–24	−0.010	−0.044 to 0.049	0.674	0.092	0.026 to 0.199	0.036	−0.015	−0.064 to 0.051	0.619
Adjusted									
Prevalence of current e‐cigarette use aged 16–24 Prevalence of current e‐cigarette use aged 16–17 Prevalence of current e‐cigarette use aged 18–24	−0.007	−0.044 to 0.053	0.786	0.097	0.013 to 0.205	0.048	−0.010	−0.059 to 0.058	0.746

CI = confidence interval; IRF = impulse response function.

The results of running SVAR models on the data over a 4‐quarter period (1 year) for those aged 16–17 are presented in eFigs 1 and 2 in the Supporting information and illustrate both the contemporaneous and cumulative impacts for the unadjusted and adjusted models, respectively.

## DISCUSSION

### Principal findings

The increase in prevalence of e‐cigarette use in England among the entire sample does not appear to have been associated with an increase in the uptake of smoking among young adults aged 16–24. In stratified analyses, large positive associations could also be ruled out for those aged 16–17 and 18– 24; however, sensitivity analyses using SVAR models could not rule out small positive associations for the youngest age group.

### Strengths and limitations

This study made use of a large and unique long‐standing monthly survey of smoking and e‐cigarette use in England. We analysed the data set using time–series models, which are particularly well‐suited to addressing our research question. We assessed the extent to which the past prevalence of the use of e‐cigarettes was able to forecast the future prevalence of a measure of smoking uptake, and established that there was little evidence of reverse causality (smoking uptake forecasting e‐cigarette use). By applying a time–series approach to population‐level estimates, we were able to directly assess the net population‐level effect of e‐cigarettes on smoking uptake in England. Our study also had several limitations. First, this study only adjusted for combined pulse effects for the tobacco control policies. This decision was based on previous research evaluating policies which have often been unable to detect sustained prolonged effects, and because there were concerns about over‐parameterization of the models. Secondly, the findings might not generalize to other countries. England has a strong tobacco control climate and a relatively liberal attitude towards e‐cigarettes. Thirdly, as with all population‐level surveys, there are issues with self‐report, including forgetting and social desirability. A recent analysis demonstrated that STS data and sales data were closely aligned, with both showing that overall cigarette sales in England have declined by almost a quarter since 2011 [20]. Fourthly, there were differences in the results for the ARIMAX and SVAR models. Although some of this discrepancy lies in the ability of SVAR models to account for feedback from the output series to the input series, there are also important model specification differences. ARIMAX models often provide better adjustment for seasonality, as they use seasonal autocorrelation terms and adjust for autoregressive and moving average autocorrelation when specifying lags [17, 37]. Fifthly, this study attempted to assess the possible evidence for a gateway effect by looking at the association between e‐cigarette use and ever regular smoking prevalence as a measure of smoking uptake. These time–series will not be affected by other important causal pathways of interest. For example, quitting smoking with the use of e‐cigarettes is not captured by the ever regular smoking variable. This study therefore only assessed gateway effects on uptake, i.e. the extent to which e‐cigarettes are associated with more or less people starting to smoke regularly, but does not assess any association with quitting (even if people were stopping successfully with e‐cigarettes they would still be reflected in estimates of ever regular smoking). The association with quitting has been assessed previously in another paper, with e‐cigarette prevalence associated with an increased prevalence of successful quit attempts [38]. While this analysis shows no negative net public health impact for the promotion of e‐cigarettes in this age group on uptake of smoking, it is therefore a conservative estimate of the potential benefits of e‐cigarette use. Finally, this study defined ever regular smoking as those using cigarettes either daily or non‐daily. This is consistent with other youth surveys in the United Kingdom (e.g. YouGov commissioned by Action on Smoking and Health and the Annual Government Survey). However, internationally there is wide variability and little consensus over the definition of regular smoking [39]. It will be important in future studies to consider the implications of this, perhaps through stratification by daily versus non‐daily smoking.

### Comparison with other studies and policy implications

The findings from this study contribute to the debate on whether e‐cigarettes could act as a gateway into smoking [3, 4, 5, 40], and are consistent with a recent population time trend analysis which showed a reduction in smoking initiation during the period of e‐cigarettes ascendance in the United States [41]. Although previous longitudinal observational studies show that there is a strong association between initiation of e‐cigarette use and later use of cigarettes [6, 7, 8], these findings may reflect a common liability whereby people with characteristics or a social environment that make them susceptible to use e‐cigarettes are also more likely to smoke cigarettes later [9, 42]. These findings are important, given the contrasting advice given by health bodies and governments in different countries. For example, Public Health England aim to minimize these risks while maximizing the public health opportunity for adult smokers to quit [43]. In contrast, the United States appears primarily focused upon what they judge to be an epidemic of youth e‐cigarette use, which threatens to engulf a new generation in nicotine addiction [44]. Research to date supports the argument that e‐cigarettes are less harmful than tobacco use, but probably cause some harm relative to never use [4, 15].

If a gateway effect exists, our results suggest that it is likely to be smaller than estimated previously [8]. If there has been a population‐level association in England, changes in e‐cigarette prevalence by 1% were probably associated with changes of ever regular smoking prevalence of less than 0.310 and 0.013 percentage points for 16–17‐ and 18–24‐year‐olds, respectively.

In some of the sensitivity analyses, there was a small positive association between e‐cigarette prevalence and ever regular smoking prevalence among 16–17‐year‐olds. However, findings are not conclusive due to possible model instability, and it remains plausible that a third unmeasured confounding variable may simultaneously be driving up ever regular smoking prevalence and e‐cigarette use in the population, creating an artificial association. For example, it may be that increasing misperceptions around the harms of e‐cigarettes are driving users into smoking [45]. Further, the most direct assessment of a gateway effect, i.e. between prevalence of e‐cigarette use among never regular smoking adolescents and ever regular smoking, showed no significant association.

Nevertheless, in so far that it was causal, the size of the association (B = 0.097) from the sensitivity analyses, and it being evident only in the youngest age group, would translate to approximately 7200 additional ever regular smokers aged 16–17 in 2018 as a consequence of e‐cigarette use. This is on the basis of assuming a prevalence of e‐cigarette use of 5% in 2018 which equates to approximately 74 000 e‐cigarette users aged 16–17 in the population (population size of those aged 16–17 ~1490 000 for 2018; i.e. 1 490 000 × 0.05 × 0.097) [46]. This needs to be weighed against the 50 000–70 000 smokers who are estimated to quit smoking each year as a consequence of using e‐cigarettes during a quit attempt [21]. These numbers are also much smaller than those proposed by previous evidence for gateway effects, which would have estimated an equivalent of 230 and 768 000 additional ever regular smokers aged 16–17 and 18–24, respectively [8].

## CONCLUSION

This time–series analysis suggests that changes in prevalence of e‐cigarette use among 16–24‐year‐olds in England does not appear to be associated with increases in the uptake of smoking in this age group. However, small associations cannot be ruled out, particularly for 16–17‐year‐olds.

## DECLARATION OF INTERESTS

E.B. and J.B. have received unrestricted research funding from Pfizer. E.B. and J.B. are funded by CRUK (C1417/A14135). L.S. has received honoraria for talks, an unrestricted research grant and travel expenses to attend meetings and workshops from Pfizer, and has acted as paid reviewer for grant awarding bodies and as a paid consultant for health‐care companies. All authors declare there are no other relationships or activities that could appear to have influenced the submitted work.

## AUTHOR CONTRIBUTIONS

**Emma Beard:** Conceptualization; formal analysis. **Jamie Brown:** Conceptualization. **Lion Shahab:** Conceptualization.

## Supporting information


**Table S1a:** Primary analysis ‐ adjusted estimated percentage point changes in ever smoking prevalence as a function of e‐cigarette prevalence, based on autoregressive integrated moving average with exogeneous input (ARIMAX) – lag on mass media (and inclusion of the affordability tobacco index)
**Table S1b:** Primary analysis ‐ adjusted estimated percentage point changes in ever smoking prevalence as a function of e‐cigarette prevalence, based on autoregressive integrated moving average with exogeneous input (ARIMAX) – no lag on mass media (and inclusion of the affordability tobacco index)
**Table S2:** Model fit indices
**Table S3:** Adjusted estimated percentage point changes in ever smoking prevalence as a function of e‐cigarette prevalence, based on Structural Vector Autoregression (SVAR) ‐ adjusted for tobacco control policies, affordability of tobacco index and tobacco control mass media spend
**Figure S1:** Unadjusted Impulse Response Function (IRF) and Cumulative IRF: impact of e‐cigarette use prevalence on ever smoking prevalence among those aged 16–17
**Figure S2:** Adjusted IRF and Cumulative IRF: impact of e‐cigarette use prevalence on ever smoking prevalence among those aged 16–17

## References

[1] Vardhan H . Why we banned E‐cigarettes: when used by never‐smokers, these can be a gateway for cigarette smoking and drug use. Times of India (TOI) 9 October, 2019.

[2] US Food Drug Administration (FDA) . Results from 2018 National Youth Tobacco Survey Show Dramatic Increase in E‐Cigarette Use Among Youth Over Past Year. Sharp Rise in E‐Cigarette Use Results in Uptick in Overall Tobacco Product Use. Silver Spring, MD: FDA; 2018.

[3] Leventhal AM , Strong DR , Kirkpatrick MG , Unger JB , Sussman S , Riggs NR , et al. Association of electronic cigarette use with initiation of combustible tobacco product smoking in early adolescence. JAMA. 2015;314:700–7. 26284721 10.1001/jama.2015.8950 PMC4771179

[4] Bauld L , MacKintosh A , Eastwood B , Ford A , Moore G , Dockrell M , et al. Young people's use of e‐cigarettes across the United Kingdom: findings from five surveys 2015–2017. Int J Environ Res Public Health. 2017;14:973. 10.3390/ijerph14090973 PMC5615510 28850065

[5] Kozlowski LT , Warner KE . Adolescents and e‐cigarettes: objects of concern may appear larger than they are. Drug Alcohol Depend. 2017;174:209–214. 29350617 10.1016/j.drugalcdep.2017.01.001

[6] Cullen KA , Ambrose BK , Gentzke AS , Apelberg BJ , Jamal A , King BA . Notes from the field: use of electronic cigarettes and any tobacco product among middle and high school students—United States, 2011–2018. Morb Mortal Wkly Rep. 2018;67:1276–1277. 10.15585/mmwr.mm6745a5 PMC6290807 30439875

[7] Berry KM , Fetterman JL , Benjamin EJ , Bhatnagar A , Barrington‐Trimis JL , Leventhal AM , et al. Association of electronic cigarette use with subsequent initiation of tobacco cigarettes in US youths. JAMA Netw Open. 2019;2:e187794‐e. 30707232 10.1001/jamanetworkopen.2018.7794 PMC6484602

[8] Soneji S , Barrington‐Trimis JL , Wills TA , Leventhal AM , Unger JB , Gibson LA , et al. Association between initial use of e‐cigarettes and subsequent cigarette smoking among adolescents and young adults: a systematic review and meta‐analysis. JAMA Pediatr. 2017;171:788–97. 28654986 10.1001/jamapediatrics.2017.1488 PMC5656237

[9] Etter JF . Gateway effects and electronic cigarettes. Addiction. 2018;113:1776–83. 28786147 10.1111/add.13924

[10] Beard E , Brown J , Michie S , Kaner E , Meier P , West R . Use of aids for smoking cessation and alcohol reduction: a population survey of adults in England. BMC Public Health. 2016;16:1237. 27931202 10.1186/s12889-016-3862-7 PMC5146832

[11] Box GE , Jenkins GM , Reinsel GC , Ljung GM . Time Series Analysis: Forecasting and Control. Hoboken, NJ: John Wiley & Sons; 2015.

[12] Hamilton JD . Time Series Analysis. Princeton, NJ: Princeton University Press; 1994.

[13] Przymus P , Hmamouche Y , Casali A , Lakhal L . Improving multivariate time series forecasting with random walks with restarts on causality graphs. New Orleans, LA: Institute of Electrical and Electronics Engineers (IEEE) International Conference on Data Mining Workshops (ICDMW); 2017, pp. 924–931. doi: 10.1109/ICDMW.2017. IEEE.

[14] Arjas E , Eerola M . On predictive causality in longitudinal studies. J Stat Plann Inference. 1993;34:361–86.

[15] Action on Smoking and Health Use of e‐cigarettes among young people in Great Britain. 2019. Available at: https://ash.org.uk/wp-content/uploads/2019/06/ASH-Factsheet-Youth-E-cigarette-Use-2019.pdf. Accessed Feb 2022.

[16] Shahab L , Brown J , Boelen L , Beard E , West R , Munafo M . Unpacking the gateway hypothesis of e‐cigarette use: the need for triangulation of individual and population level data. Nicotine Tob Res. 2022. 10.1093/ntr/ntac035 PMC9278819 35137222

[17] Beard E , Marsden J , Brown J , Tombor I , Stapleton J , Michie S , et al. Understanding and using time–series analyses in addiction research. Addiction. 2019;114:1866–84. 31058392 10.1111/add.14643

[18] Beard E , Dienes Z , Muirhead C , West R . Using Bayes factors for testing hypotheses about intervention effectiveness in addictions research. Addiction. 2016;111:2230–47. 27347846 10.1111/add.13501 PMC5111611

[19] Fidler JA , Shahab L , West O , Jarvis MJ , McEwen A , Stapleton JA , et al. ‘The smoking toolkit study’: a national study of smoking and smoking cessation in England. BMC Public Health. 2011;11:479. 21682915 10.1186/1471-2458-11-479 PMC3145589

[20] Jackson SE , Beard E , Kujawski B , Sunyer E , Michie S , Shahab L , et al. Comparison of trends in self‐reported cigarette consumption and sales in England, 2011 to 2018. JAMA Netw Open. 2019;2:e1910161‐e. 31461148 10.1001/jamanetworkopen.2019.10161 PMC6716287

[21] Beard E , West R , Michie S , Brown J . Association of prevalence of electronic cigarette use with smoking cessation and cigarette consumption in England: a time‐series analysis between 2006 and 2017. Addiction. 2021;115:961–74. 10.1111/add.14851 PMC7187187 31621131

[22] Tombor I , Beard E , Brown J , Shahab L , Michie S , West R . Randomized factorial experiment of components of the SmokeFree baby smartphone application to aid smoking cessation in pregnancy. Transl Behav Med. 2019;9:583–93. 30011020 10.1093/tbm/iby073 PMC6629841

[23] Slater SJ , Chaloupka FJ , Wakefield M , Johnston LD , O'Malley PM . The impact of retail cigarette marketing practices on youth smoking uptake. Arch Pediatr Adolesc Med. 2007;161:440–5. 17485618 10.1001/archpedi.161.5.440

[24] Owusu‐Dabo E , Lewis S , McNeill A , Gilmore A , Britton J . Smoking uptake and prevalence in Ghana. Tob Control. 2009;18:365–70. 19581276 10.1136/tc.2009.030635 PMC2745559

[25] Beard EV , West R , Jarvis M , Michie S , Brown J . ‘S’‐shaped curve: modelling trends in smoking prevalence, uptake and cessation in Great Britain from 1973 to 2016. Thorax. 2019;74:875–81. 31391317 10.1136/thoraxjnl-2018-212740 PMC6824614

[26] Edwards R , Carter K , Peace J , Blakely T . An examination of smoking initiation rates by age: results from a large longitudinal study in New Zealand. Aust NZ J Public Health. 2013;37:516–9. 10.1111/1753-6405.12105 24892149

[27] Health and Social Care Information Centre . NHS Stop Smoking Services Collection 2015. Available at: www.hscic.gov.uk/stopsmoking

[28] Hackshaw L , McEwen A , West R , Bauld L . Quit attempts in response to smoke‐free legislation in England. Tob Control. 2010;19:160–4. 20378592 10.1136/tc.2009.032656

[29] Beard E , Michie S , Fidler J , West R . Use of nicotine replacement therapy in situations involving temporary abstinence from smoking: a national survey of English smokers. Addict Behav. 2013;38:1876–9. 23313941 10.1016/j.addbeh.2012.09.013

[30] Fidler JA , West R . Changes in smoking prevalence in 16–17‐year‐old versus older adults following a rise in legal age of sale: Findings from an English population study. Addiction. 2010;105:1984–8. 20722633 10.1111/j.1360-0443.2010.03039.x

[31] European Commission . Directive 2014/40/EU of the European Parliament and of the Council. Brussels, Belgium: European Commission; 2014.

[32] NHS Digital . Statistics on Smoking—England, 2018 [PAS]. Leeds, UK: NHS Digital; 2018.

[33] Kuipers MA , Partos T , McNeill A , Beard E , Gilmore AB , West R , et al. Smokers’ strategies across social grades to minimise the cost of smoking in a period with annual tax increases: evidence from a national survey in England. BMJ Open. 2019;9:e026320. 10.1136/bmjopen-2018-026320 PMC6597620 31243031

[34] Regan AK , Promoff G , Dube SR , Arrazola R . Electronic nicotine delivery systems: adult use and awareness of the ‘e‐cigarette’ in the USA. Tob Control. 2013;22:19–23. 22034071 10.1136/tobaccocontrol-2011-050044

[35] Von Elm E , Altman DG , Egger M , Pocock SJ , Gøtzsche PC , Vandenbroucke JP . Strengthening The Reporting of Observational studies in Epidemiology (STROBE) statement: guidelines for reporting observational studies. BMJ. 2007;335:806–8. 17947786 10.1136/bmj.39335.541782.AD PMC2034723

[36] Ly A , Verhagen J , Wagenmakers E‐J . Harold Jeffreys's default Bayes factor hypothesis tests: explanation, extension, and application in psychology. J Math Psychol. 2016;72:19–32.

[37] Pfaff B . VAR, SVAR and SVEC models: implementation within R package vars. J Stat Softw. 2008;27:1–32.

[38] Beard E , West R , Michie S , Brown J . Association between electronic cigarette use and changes in quit attempts, success of quit attempts, use of smoking cessation pharmacotherapy, and use of stop smoking services in England: time–series analysis of population trends. BMJ. 2016;354:i4645. 27624188 10.1136/bmj.i4645

[39] Husten CG . How should we define light or intermittent smoking? Does it matter? Nicotine Tob Res. 2009;11:111–21. 19246425 10.1093/ntr/ntp010 PMC2658911

[40] National Academies of Sciences, Engineering and Medicine . Public Health Consequences of e‐cigarettes. London, UK: National Academies Press; 2018. 29894118

[41] Levy DT , Warner KE , Cummings KM , Hammond D , Kuo C , Fong GT , et al. Examining the relationship of vaping to smoking initiation among US youth and young adults: a reality check. Tob Control. 2019;28:629–35. 30459182 10.1136/tobaccocontrol-2018-054446 PMC6860409

[42] Kim S , Selya AS . The relationship between electronic cigarette use and conventional cigarette smoking is largely attributable to shared risk factors. Nicotine Tob Res. 2019;22:1123–30. 10.1093/ntr/ntz157 PMC7291806 31680169

[43] Dockrell M . 2019. Available at: https://publichealthmatters.blog.gov.uk/2019/02/27/e-cigarette-evidence-update-patterns-and-use-in-adults-and-young-people/

[44] Food and Drug Administration (FDA) . Results from 2018 National Youth Tobacco Survey show dramatic increase in e‐cigarette use among youth over past year [press release]. Silver Spring, MD: FDA; 2018.

[45] McNeill A , Brose LS , Calder R , Bauld L , Robson D . Evidence Review of e‐cigarettes and Heated Tobacco Products 2018. A report commissioned by Public Health England. London, UK: Public Health England; 2018.

[46] Office for National Statistics (ONS) . Population Estimates—annual population estimates for the UK and its constituent countries, the regions and counties of England, and local authorities and their equivalents. Estimates for lower and middle layer Super Output Areas, Westminster parliamentary constituencies, electoral wards and National Parks in England and Wales and clinical commissioning groups in England. Newport, UK: ONS; 2017.

