# Supplementary material for: Association of quarterly prevalence of e‐cigarette use with ever regular smoking among young adults in England: a time–series analysis between 2007 and 2018
Source: Addiction. 2022 Mar 9;117(8):2283–93. doi: 10.1111/add.15838 (PMC9543274; doi:10.1111/add.15838)
Supplement: Supplementary file 1 — Table S1a: Primary analysis ‐ adjusted estimated percentage point changes in ever smoking prevalence as a function of e‐cigarette prevalence, based on autoregressive integrated moving average with exogeneous input (ARIMAX) – lag on mass media (and inclusion of the affordability tobacco index) Table S1b: Primary analysis ‐ adjusted estimated percentage point changes in ever smoking prevalence as a function of e‐cigarette prevalence, based on autoregressive integrated moving average with exogeneous input (ARIMAX) – no lag on mass media (and inclusion of the affordability tobacco index) Table S2: Model fit indices Table S3: Adjusted estimated percentage point changes in ever smoking prevalence as a function of e‐cigarette prevalence, based on Structural Vector Autoregression (SVAR) ‐ adjusted for tobacco control policies, affordability of tobacco index and tobacco control mass media spend Figure S1: Unadjusted Impulse Response Function (IRF) and Cumulative IRF: impact of e‐cigarette use prevalence on ever smoking prevalence among those aged 16–17 Figure S2: Adjusted IRF and Cumulative IRF: impact of e‐cigarette use prevalence on ever smoking prevalence among those aged 16–17 [file ADD-117-2283-s001.docx]

**Supplementary Table 1a: Primary analysis - adjusted estimated percentage point changes in ever smoking prevalence as a function of e-cigarette prevalence, based on autoregressive integrated moving average with exogeneous input (ARIMAX) – lag on mass media (and inclusion of the affordability tobacco index)**

|  | **Adjusted** | | | | | | | | |
| --- | --- | --- | --- | --- | --- | --- | --- | --- | --- |
|  | **Prevalence of ever smoking 16-24^a^** | | | **Prevalence of ever smoking 16-17^b^** | | | **Prevalence of ever smoking 18-24^c^** | | |
|  | **Percentage change per 1% change in the exposure** | **95% CI** | **Percentage change per 1% change in the exposure** | **Percentage change per 1% change in the exposure** | **95% CI** | **P value** | **Percentage change per 1% change in the exposure** | **95% CI** | **P value** |
| Prevalence of current e-cigarette use 16-24  Prevalence of current e-cigarette use 16-17  Prevalence of current e-cigarette use 18-24 | 0.009 | -0.013 to  0.031 | 0.421 | 0.082 | 0.011 to 0.152 | 0.023 | -0.007 | -0.020 to 0.033 | 0.614 |
| *Tobacco control mass media spend (lag 1)*  *Affordability of tobacco index* | 0.017  0.082 | 0.011 to  0.023  -0.703 to  0.867 | <0.001  0.838 | 0.026  -1.054 | -0.005 to 0.058  -3.287 to 1.179 | 0.105  0.355 | 0.013  0.093 | 0.005 to 0.020  -0.783 to 0.970 | 0.001  0.835 |
|  | **Total change due to the exposure** | **95% CI** | **P value** | **Total change due to the exposure** | **95% CI** | **P value** | **Total change due to the exposure** | **95% CI** | **P value** |
| *Tobacco control policies* | **0.047** | **-0.009 to 0.102** | **0.098** | **0.0.82** | **-0.151 to 0.316** | **0.488** | **0.037** | **-0.029 to 0.102** | **0.273** |
| Model  Adjusted R-squared | ARIMA(0,1,1)(0,0,0)_4_  **0.79** |  |  | ARIMA(0,1,1)(0,0,0)_4_  **0.50** |  |  | ARIMA(0,1,1)(0,0,0)_4_  **0.73** |  |  |
| Bayes Factor (Robustness region) | **0.001 (-**∞ to **-0.011** $\boldsymbol{\cap}$**0.036 to** ∞) | |  | **0.047 (-**∞ to **-0.018** $\boldsymbol{\cap}$**0.064 to** ∞) | |  | **0.001 (-**∞ to **-0.034** $\boldsymbol{\cap}$**0.017 to** ∞) | |  |

**Supplementary Table 1b: Primary analysis - adjusted estimated percentage point changes in ever smoking prevalence as a function of e-cigarette prevalence, based on autoregressive integrated moving average with exogeneous input (ARIMAX) – no lag on mass media (and inclusion of the affordability tobacco index)**

|  | **Adjusted** | | | | | | | | |
| --- | --- | --- | --- | --- | --- | --- | --- | --- | --- |
|  | **Prevalence of ever smoking 16-24^a^** | | | **Prevalence of ever smoking 16-17^b^** | | | **Prevalence of ever smoking 18-24^c^** | | |
|  | **Percentage change per 1% change in the exposure** | **95% CI** | **Percentage change per 1% change in the exposure** | **Percentage change per 1% change in the exposure** | **95% CI** | **P value** | **Percentage change per 1% change in the exposure** | **95% CI** | **P value** |
| Prevalence of current e-cigarette use 16-24  Prevalence of current e-cigarette use 16-17  Prevalence of current e-cigarette use 18-24 | -0.006 | -0.037 to 70.026 | 0.728 | 0.078 | 0.010 to 0.146 | 0.025 | -0.006 | -0.033 to 0.021 | 0.658 |
| *Tobacco control mass media spend*  *Affordability of tobacco index* | 0.018  -0.393 | -0.001 to  0.038  -0.870 to  0.084 | 0.067  0.106 | 0.003  -1.651 | -0.070 to 0.075  -2.304 to -0.999 | 0.945  <0.001 | 0.022  -0.440 | 0.001 to 0.043  -0.718 to -0.163 | 0.040  0.002 |
|  | **Total change due to the exposure** | **95% CI** | **P value** | **Total change due to the exposure** | **95% CI** | **P value** | **Total change due to the exposure** | **95% CI** | **P value** |
| *Tobacco control policies* | **0.067** | **0.006 to 0.128** | **0.031** | **0.134** | **-0.102 to 0.370** | **0.265** | **0.069** | **0.001 to 0.136** | **0.046** |
| Model  Adjusted R-squared | ARIMA(0,1,1)(0,0,0)_4_  **0.70** |  |  | ARIMA(0,1,1)(0,0,0)_4_  **0.47** |  |  | ARIMA(0,1,1)(0,0,0)_4_  **0.70** |  |  |
| Bayes Factor (Robustness region) | **0.001 (-**∞ to **-0.036** $\boldsymbol{\cap}$**0.021 to** ∞) | |  | **0.042 (-**∞ to **-0.018** $\boldsymbol{\cap}$**0.580 to** ∞) | |  | **0.001 (-**∞ to **-0.032** $\boldsymbol{\cap}$**0.017 to** ∞) | |  |

**Supplementary Table 2: Model fit indices**

|  | **Age 16-24** | **Age 16-17** | **Age 18-24** |
| --- | --- | --- | --- |
|  | **AIC** | | |
| Unadjusted model - Table 2 | -103.70 | 21.77 | -95.15 |
| Adjusted model - Table 3 | -105.77 | 25.30 | -95.79 |
| Adjusted model with affordability – Supplementary Table 1 | -105.61 | 18.46 | -98.37 |

Note: AIC - Akaike information criterion (AIC); models with AIC differences relative to the unadjusted model (delta $\Delta$) of <2 have little support, while >4 have substantial support; lower AIC = better model fit ^1^.

**Supplementary Table 3: Adjusted estimated percentage point changes in ever smoking prevalence as a function of e-cigarette prevalence, based on Structural Vector Autoregression (SVAR) - adjusted for tobacco control policies, affordability of tobacco index and tobacco control mass media spend**

|  | **Prevalence of ever smoking 16-24** | | | **Prevalence of ever smoking 16-17** | | | **Prevalence of ever smoking 18-24** | | |
| --- | --- | --- | --- | --- | --- | --- | --- | --- | --- |
|  | **IRF**  **Percentage change per 1% change in the exposure** | **95% CI** | **P value** | **IRF**  **Percentage change per 1% change in the exposure** | **95% CI** | **P value** | **IRF**  **Percentage change per 1% change in the exposure** | **95% CI** | **P value** |
|  |  |  |  |  |  |  |  |  |  |
| Prevalence of current e-cigarette use 16-24  Prevalence of current e-cigarette use 16-17  Prevalence of current e-cigarette use 18-24 | -0.007 | **-0.043 to**  **0.053** | **0.754** | **0.098** | **0.008 to 0.197** | **0.042** | -0.013 | **-0.062 to 0.058** | **0.681** |


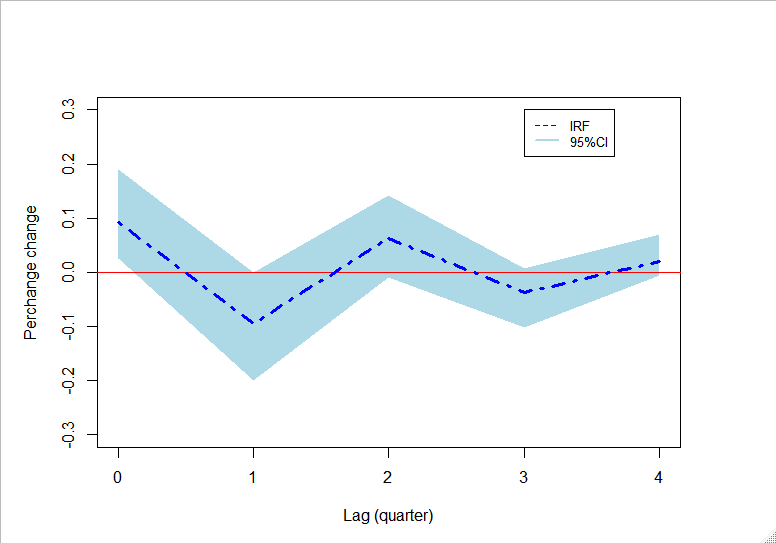


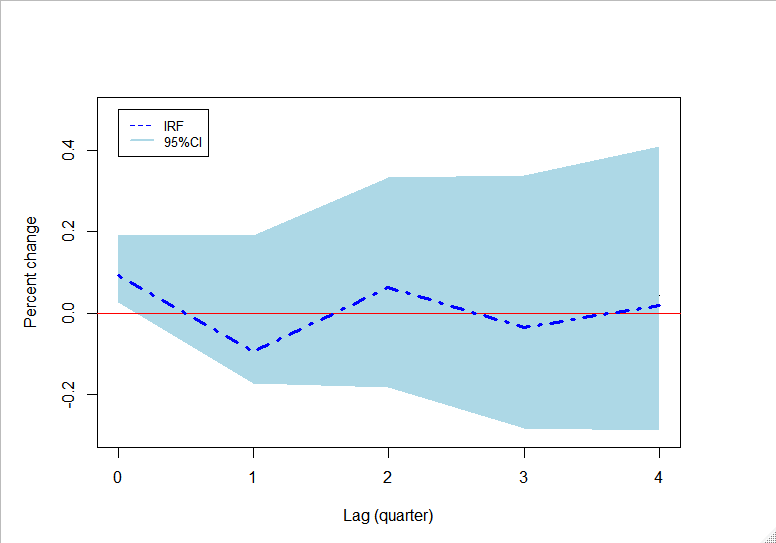


**Supplementary Figure 1: Unadjusted Impulse Response Function (IRF) and Cumulative IRF: impact of e-cigarette use prevalence on ever smoking prevalence among those aged 16-17**


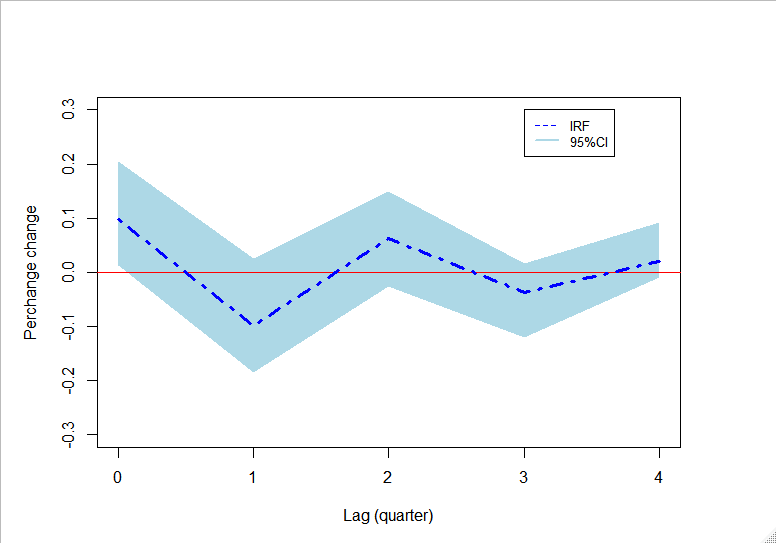


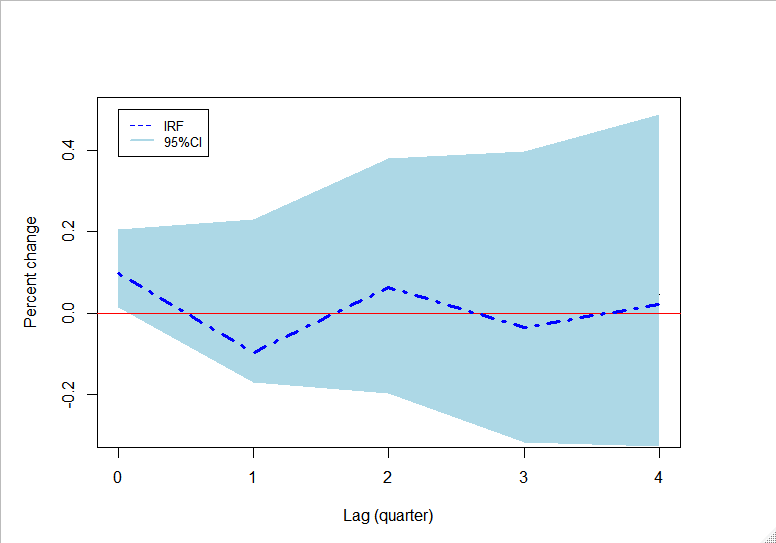


**Supplementary Figure 2: Adjusted IRF and Cumulative IRF: impact of e-cigarette use prevalence on ever smoking prevalence among those aged 16-17**

**Supplementary Analysis**

The analysis plan was pre-registered on the Open Science Framework (osf.io/zu235). Data were aggregated quarterly and were weighted to match the population profile in England on age, social grade, region, tenure, ethnicity, and working status within sex. The dimensions are derived from a combination of the 2011 census , Office for National Statistics, and an annual random probability survey conducted for the National Readership Survey ^2^. All data were analysed in R ^3^. The primary analysis focused on the association between e-cigarette prevalence and ever smoking prevalence among those aged 16 to 24, with an additional stratified analysis with those aged 16 to 17 and 18 to 24.

**ARIMAX approach**

Autoregressive Integrated Moving Average with Exogeneous Input (ARIMAX) analysis was used with the TSA package in R ^4^. ARIMAX is an extension of autoregressive integrated moving average analysis (ARIMA), which produces forecasts based upon prior values in the time series analysis (AR terms) and the errors made by previous predictions (MA terms). Both adjusted and unadjusted models are reported in this paper.

Standard recommended procedures were used to select the ARIMAX models ^5,6^. First, we assessed each time series analysis for outlying values (using box plots) which may bias the results and the presence of exogeneity using the Granger Causality test. Secondly, plots of the differenced data and unit root tests (i.e. Osborn-Chui-Smith-Birchenhall test (OCBS) and Kwiatkowski, Phillips, Schmidt, and Shin (KPSS) test) were used to determine the number of seasonal and non-seasonal differences required for the time series analyses to be made stationary ^7^. This was confirmed by the Augmented Dickey-Fuller test (ADF). To identify the most appropriate transfer function for the continuous explanatory variables (i.e. to identify the manner in which past values of the e-cigarette time series analysis are used to forecast future values of the outcome) the sample cross-correlation function was checked for each ARIMAX model, with pre-whitened data ^8^. Pre-whitening removes autocorrelation in the input series that may cause spurious cross-correlation effects. Additional checks were also run by comparing univariate ARIMAX models with variations for the transfer function.

Next, in order to determine the initial values of the AR and MA terms for the baseline models, the autocorrelation function (ACF) and partial autocorrelation function (PACF) were assessed. Additional models with various fitted AR and MA terms were then compared to this baseline model using the Akaike Information Criterion (AIC). According to the Box-Jenkins method, in ARIMA (p, d, q) the value of p and q should be 2 or less or the total number of parameters should be less than 3 ^5^. Therefore, we only checked ARIMAX models for p and q values of 3 or fewer. The models with lower AIC values were selected. Finally, the ACF for the residuals of the best fitting models were checked for additional correlation (thus the need for additional MA/AR seasonal or non-seasonal terms) and the coefficients of the correlation terms assessed for significance and whether they fall within the bounds of stationarity and invertibility ^9^. The Ljung-Box test for white noise and plot of the ACF for model residuals were also used to statistically evaluate the degree to which the residuals are free of serial correlation ^10^, and the final model residuals assessed for normality. There was some deviation from the assumption of normally distributed residuals for the model assessing e-cigarette prevalence and ever smoking among those aged 16 to 17.

Outliers were detected for ever smoking prevalence and tobacco control mass media expenditure. None of these outliers were deemed to be erroneous values and so they were not excluded from the analysis. Generally, outliers are only justifiably removed if they are determined to be errors and they fall at the start or end of the series ^11^. The assumption of weak exogeneity (i.e., Y can depend on the lagged values of X but the reverse must not be true) was met for most series. There was some evidence for the violation of the assumption of weak exogeneity between affordability and ever smoking.

**Bayes factors**

Bayes factors were calculated for non-significant findings in R using code described by Dienes ^12^. This helps to determine if there is evidence for the null hypothesis of no difference or the data are insensitive to detect an effect. This approach requires the specification of an expected effect size (i.e. a plausible range of predicted values based on previous studies, judgement or clinical significance), the published effect size (e.g. risk difference) and SE of this parameter. It assumes that the sampling distribution of the parameter estimate is Gaussian.

The expected effect size corresponded to a relative risk of 3.1. This was on the basis of a recent meta-analysis of seven cohort studies in youths which reported that baseline ever e-cigarette use strongly predicted cigarette smoking initiation with an Odds Ratio of 3.5 (Relative Risk of ~3.1 assuming a baseline prevalence of smoking of 4.6%). This would be interpreted in terms of elasticity if a log transformation is applied as a 1% increase in the point prevalence of e-cigarette use would be associated with a 3.1% increase in the point prevalence of ever smoking.

A Gaussian distribution was specified where the population parameter values close to the mean are assumed to be more plausible than others. A default standard deviation of mean/2 was used ^13^. Bayes factors are interpreted based on Jeffreys’ cut-offs ^14^, which indicate the strength of evidence for or against the null hypothesis. We also calculated a robustness region for each Bayes factor. The robustness region is a range of expected effect sizes that lead to the same qualitative conclusion (i.e. good evidence for the alternative hypothesis if BF > 3; good evidence for the null hypothesis if BF < 1/3; and largely insensitive otherwise) ^12^.

**Sensitivity analyses**

***Testing the robustness of data assumptions***

We conducted two sensitivity analyses to test the assumption that e-cigarette use among respondents who were never smokers and long-term ex-smokers was zero before October 2013, which was when questions were first asked of the entire sample. In the first analysis, we adjusted the ARIMAX model for a step level change in October 2013. Secondly, we used a ratio of use among long-term ex-smokers and never smokers versus past-year smokers for the 1st year of assessment [0.0026=(0.5%/18.9%)/100] to adjust the prevalence prior to October 2013. This was done by assuming the number of long-term ex-smokers and never smokers using e-cigarettes is the same as the proportion of past year smokers who use e-cigarettes corrected by the calculated ratio.

Two sensitivity analyses were also conducted to assess the assumption that prevalence of e-cigarette use between January 2007 and June 2011 when questions on e-cigarettes were not asked was 0.1%. The first analysis instead assumed a linear function starting at close to zero from 2007 (0.001) ending at the quarter 2 estimate for 2012, while the second used Kalman Smoothing for univariate time series to impute the values ^15^.

***E-cigarette use among never smokers***

One sensitivity analysis changed the input variable to e-cigarette prevalence among never smokers only.

***Accounting for feedback from prevalence of ever smoking***

Due to evidence of violation of the assumption of weak exogeneity for affordability and it being theoretically plausible that ever smoking may ‘causally’ affect prevalence of e-cigarette use, additional structural time-series models known as Structural Vector Autoregression (SVAR) were run. SVAR is an economic method that allows for the estimation of the effects and impacts of a number of different time series on each other while accounting for any residual feedback from the output series to the input series. It does this, by placing restrictions on the coefficients in the model and therefore incorporates contemporaneous effects between the series ^16,17^.

We first tested the assumption of no co-integrating relationships using the Johansen Test for Cointegrating Time Series ^18^. Such relationships occur when two variables share a common trend, and thus a linear combination of the variables is stationary. As with the ARIMA models, prior to estimation, the mean and variance properties of the series were checked to look for non-stationarity, with data log transformed and differenced. Seasonality was then modelled directly in the SVAR model using a dummy variable. SVAR estimation requires a lag structure for the autoregressive autocorrelation to be specified for the model. To select the most appropriate lag we compared the AIC (as the primary measure of fit), the Schwarz Bayesian Information Criterion (SIC) and the Hannan–Quinn Information Criterion. An autoregressive autocorrelation of lag-1 was identified. To ensure that the final model had normally distributed and white noise (or random) residuals, we conducted tests of residual autocorrelation and skewness.

The results, unlike with ARIMA, do not follow those from a standard regression model. They are presented using impulse response functions (IRF) and cumulative impulse response functions (CIRF). These functions indicate the response of a series when it has been impacted on by another series and plots the accumulation of such shocks over a specified period of time. Thus, in this case, the IRFs shows the impact of a sudden increase in the use of e-cigarettes among 16-17 year olds on ever smoking prevalence among 16-17 year olds over a 4 quarter period (one year). SVAR models report bootstrapped confidence intervals.

Given the findings in the primary analysis, we also ran two additional sensitivity analyses whereby the association of e-cigarette use only among never smokers with ever smoking was assessed among those aged 16-17 and 18-24.

**Unregistered amendments to the analysis plan**

In the primary and sensitivity analyses, it was evident that the coefficient for mass media was implausible, i.e. increases in expenditure on mass media were associated with increases in ever smoking. We could not identify a theoretical reason to explain this, given the strong evidence that it promotes successful attempts to stop ^19^. Various additional models were run to identify if there was a model specification problem. The positive association with mass media was not evident when the affordability of tobacco index was removed from the model as a covariate. Removal of the affordability index did not substantially decrease model fit for 16-24 year olds (see Supplementary Table 2). Model fit was improved with the inclusion of the affordability index for 16-17 and 18-24 year olds, but this may be due to the AIC having a tendency to select overparameterized models for more volatile time series based on small samples ^1^. There was also evidence of high multi-collinearity between affordability and the other variables in the model, in particular with e-cigarette prevalence. VIFs for affordability ranged from 2.14 to 5.29, with thresholds for price elasticity often set to <2 ^20^. VIFs for the other covariates were all <1.2. We therefore decided to remove affordability from the main adjusted model. A lag of 1 was also identified for mass media, but this also led to sign flipping and an over parameterised model and so no lags were entered into the final model. The findings with the adjustment for affordability and lag for mass media can be found in Supplementary Table 1a and 1b.

**References**

1. Burnham KP, Anderson DR. Model selection and. 2002.

2. Fidler JA, Shahab L, West O, et al. 'The smoking toolkit study': a national study of smoking and smoking cessation in England. *BMC Public Health.* 2011;11(1):479.

3. R Development Core Team. R: A language and environment for statistical computing. R foundation for statistical computing, Vienna, Austria. <http://www.R-project.org>. Published 2008. Accessed.

4. Chan K-S, Ripley B, Chan MK-S, Chan S. Package ‘TSA’. 2018.

5. Box GE, Jenkins GM, Reinsel GC. *Time-series analysis: forecasting and control.* Vol 734: John Wiley & Sons; 2011.

6. Box GE, Tiao GC. Intervention analysis with applications to economic and environmental problems. *Journal of the American Statistical association.* 1975;70(349):70-79.

7. Phillips PC, Perron P. Testing for a unit root in time-series regression. *Biometrika.* 1988;75(2):335-346.

8. Cryer JD, Chan K-S. *Time-series analysis - with applications in R.* London: Springer-Verlag New York; 2008.

9. Yaffee RA, McGee M. *An introduction to time-series analysis and forecasting: with applications of SAS® and SPSS®.* Elsevier; 2000.

10. Montgomery DC, Jennings CL, Kulahci M. *Introduction to time-series analysis and forecasting.* John Wiley & Sons; 2015.

11. Beard E, Marsden J, Brown J, et al. Understanding and using time series analyses in addiction research. *Addiction.* 2019;114(10):1866-1884.

12. Dienes Z. How do I know what my theory predicts?. . 2019.

13. Dienes Z. Using Bayes to get the most out of non-significant results. *Frontiers in psychology.* 2014;5.

14. Jeffreys H. *The theory of probability.* OUP Oxford; 1998.

15. Moritz S, Bartz-Beielstein T. imputeTS: time series missing value imputation in R. *The R Journal.* 2017;9(1):207-218.

16. Pfaff B. VAR, SVAR and SVEC models: Implementation within R package vars. *Journal of Statistical Software.* 2008;27(4):1-32.

17. Kilian L, Lütkepohl H. *Structural vector autoregressive analysis.* Cambridge University Press; 2017.

18. Muscatelli VA, Hurn S. Cointegration and dynamic time series models. *Journal of Economic Surveys.* 1992;6(1):1-43.

19. Kuipers MA, Beard E, West R, Brown J. Associations between tobacco control mass media campaign expenditure and smoking prevalence and quitting in England: a time series analysis. *Tobacco control.* 2018;27(4):455-462.

20. Chatterjee S, Hadi AS. *Regression analysis by example.* John Wiley & Sons; 2015.
